# Causal Role of Alcohol Consumption in an Improved Lipid Profile: The Atherosclerosis Risk in Communities (ARIC) Study

**DOI:** 10.1371/journal.pone.0148765

**Published:** 2016-02-05

**Authors:** Khanh N. Vu, Christie M. Ballantyne, Ron C. Hoogeveen, Vijay Nambi, Kelly A. Volcik, Eric Boerwinkle, Alanna C. Morrison

**Affiliations:** 1 School of Public Health, University of Texas Health Science Center at Houston, Houston, Texas, United States of America; 2 Section of Cardiovascular Research, Baylor College of Medicine, Houston, Texas, United States of America; 3 Houston Methodist Debakey Heart and Vascular Center, Houston, Texas, United States of America; 4 Michael E DeBakey Veterans Affairs Hospital, Houston, Texas, United States of America; 5 Department of Biochemistry and Molecular Biology, University of Texas Medical School at Houston, Houston, Texas, United States of America; 6 The Human Genome Sequencing Center, Baylor College of Medicine, Houston, Texas, United States of America; Innsbruck Medical University, AUSTRIA

## Abstract

**Introduction:**

Health benefits of low-to-moderate alcohol consumption may operate through an improved lipid profile. A Mendelian randomization (MR) approach was used to examine whether alcohol consumption causally affects lipid levels.

**Methods:**

This analysis involved 10,893 European Americans (EA) from the Atherosclerosis Risk in Communities (ARIC) study. Common and rare variants in alcohol dehydrogenase and acetaldehyde dehydrogenase genes were evaluated for MR assumptions. Five variants, residing in the *ADH1B*, *ADH1C*, and *ADH4* genes, were selected as genetic instruments and were combined into an unweighted genetic score. Triglycerides (TG), total cholesterol, high-density lipoprotein cholesterol (HDL-c) and its subfractions (HDL2-c and HDL3-c), low-density lipoprotein cholesterol (LDL-c), small dense LDL-c (sdLDL-c), apolipoprotein B (apoB), and lipoprotein (a) (Lp(a)) levels were analyzed.

**Results:**

Alcohol consumption significantly increased HDL2-c and reduced TG, total cholesterol, LDL-c, sdLDL-c, and apoB levels. For each of these lipids a non-linear trend was observed. Compared to the first quartile of alcohol consumption, the third quartile had a 12.3% lower level of TG (p < 0.001), a 7.71 mg/dL lower level of total cholesterol (p = 0.007), a 10.3% higher level of HDL2-c (p = 0.007), a 6.87 mg/dL lower level of LDL-c (p = 0.012), a 7.4% lower level of sdLDL-c (p = 0.037), and a 3.5% lower level of apoB (p = 0.058, p_overall_ = 0.022).

**Conclusions:**

This study supports the causal role of regular low-to-moderate alcohol consumption in increasing HDL2-c, reducing TG, total cholesterol, and LDL-c, and provides evidence for the novel finding that low-to-moderate consumption of alcohol reduces apoB and sdLDL-c levels among EA. However, given the nonlinearity of the effect of alcohol consumption, even within the range of low-to-moderate drinking, increased consumption does not always result in a larger benefit.

## Introduction

Low-to-moderate alcohol consumption has been associated with cardiovascular health benefits in observational [1–3] and experimental studies [4,5], but the mechanism is still unclear. Alcohol consumption may introduce a cardiovascular benefit by improving an individual’s lipid profile, including an effect on HDL-c levels, HDL particle concentration, and HDL-c subfractions [6,7]. The relationship between low-to-moderate alcohol use and LDL-c or TG is less clear, with studies reporting reduced LDL-c or TG levels [8–15], no effect [4], or a worsened blood lipid profile [5,13,16].

The observed association between alcohol use and lipids can be confounded by demographic, social and behavioral factors, as well as access to health care, and health-related conditions [17,18]. Mendelian randomization (MR) studies using instrumental variable (IV) analysis and genetic instruments can facilitate causal inference in observational studies by reducing the issues of residual confounding and reverse causation [19–27]. Using genetic variants that influence alcohol consumption may better capture the role of life-long alcohol use [28]. A limited number of MR studies have been conducted to evaluate the relationship between alcohol consumption and lipid levels, and the results have been largely inconclusive [2,28,29]. There is no previous MR study investigating the causal role of alcohol consumption on HDL-c subfractions, HDL2-c and HDL3-c. Evaluation of these subfractions may provide a more complete picture, as HDL is highly heterogeneous in terms of particle size, lipid component, and functionality and it is hypothesized that not all HDL subclasses have anti-atherogenic effects [30]. The relationship between alcohol consumption and sdLDL-c, apoB, and Lp(a) has also not been previously investigated. This MR study aims to investigate the causal link between low-to-moderate alcohol consumption and blood levels of TG, total cholesterol, HDL-c, HDL2-c, HDL3-c, LDL-c, sdLDL-c, apoB, and Lp(a) among European Americans (EAs).

## Methods

This study involved 10,893 EAs from the Atherosclerosis Risk in Communities (ARIC) study, an ongoing prospective cohort in four communities in the US: Forsyth County, North Carolina; Washington County, Maryland; Minneapolis suburbs, Minnesota; and Jackson, Mississippi. A description of the ARIC study objectives, design, and procedures is provided elsewhere [31]. Briefly, ARIC participants received an extensive baseline examination (1987–1989), including collection of medical, social, and demographic data. Three follow-up examinations were performed at three-year intervals, and a fifth exam was conducted in 2011 to 2013. Participant follow-up also occurred annually, by telephone, to maintain contact and to assess health and vital status of the cohort. Individuals were categorized as EAs by self-report. The ARIC study has been approved by Institutional Review Boards (IRB) at all participating institutions: University of North Carolina at Chapel Hill IRB, Johns Hopkins University IRB, University of Minnesota IRB, and University of Mississippi Medical Center IRB. Study participants provided written informed consent at all study visits.

This study focused on self-reported alcohol consumption at two of the examinations, baseline and visit 4, during which the lipid levels included in this study were measured. Participants were interviewed in person using a dietary questionnaire, and they were asked if they currently or formerly drank alcoholic beverages. For current drinkers, information about the frequency and amount of wine, beer, or hard liquor consumption was collected. The amount of alcohol consumed in grams per week (g/wk) was calculated with the estimate that 4 oz wine was equal to 10.8 g, 12 oz beer was equal to 13.2 g, and 1.5 oz liquor was equal to 15.1 g ethanol. Alcohol consumption was recorded as 0 g/wk for current drinkers having less than one drink per week. Total alcohol consumption was analyzed as the natural log of (alcohol use in g/wk +1) given the skewed distribution. Current drinkers were further classified as infrequent drinkers if they had less than one drink per week, as low-to-moderate drinkers if they drank ≤ 210 g/wk for men and ≤ 105 g/wk for women, and were classified as heavy drinkers if they drank > 210 g/wk for men and > 105 g/wk for women [32–35].

TG, total cholesterol, HDL-c and its subfractions, LDL-c, and Lp(a) were measured from 12-hour fasting blood samples collected at baseline. Plasma total cholesterol [36] and TG [37] were measured by enzymatic methods, with the use of reagents supplied by Boehringer-Mannheim Biochemical, and were adapted for analysis in the Cobas-Bioanalyzer (Roche). HDL-c level was determined by measuring cholesterol in the supernate after plasma precipitation with MgCl_2_ and dextran sulfate according to the method of Warnick et al [38]. HDL3-c level was determined after reprecipitation of the total HDL-c supernate with different concentrations of MgCl_2_ and dextran sulfate. HDL2-c level was calculated by subtracting the HDL3-c value from the value of total HDL-c [39]. LDL-c was calculated from the levels of total cholesterol, HDL-c, and TG by the Friedewald formula [40]. LDL-c was not determined in individuals with plasma TG levels >400 mg/dL [41]. Lp(a) was measured as total protein component (apolipoprotein A plus apoB) with a double-antibody ELISA technique for apolipoprotein A detection [42]. Since Lp(a) assay used in ARIC at baseline could be sensitive to apo(a) isoform size, we performed a correction, multiplying by a factor of 1.326, to match with a newer Lp(a) assay that is insensitive to apo(a) isoform size when calibrated with the International Federation of Clinical Chemistry proposed reference material in molar units [43]. SdLDL-c and apoB were measured from 12-hour fasting blood samples collected at visit 4. SdLDL-c was directly measured by a homogeneous assay method (sd-LDL-EX “Seiken”, Denka Seiken, Tokyo, Japan) on a Hitachi 917 automated chemistry analyzer [44]. ApoB was measured by an immunonephelometric assay using a BNII nephelometer (Siemens Healthcare Diagnostics, Deerfield, IL) [44]. Due to the skewed distribution of TG, HDL-c, HDL2-c, sdLDL-c, apoB, and Lp(a), these measures were evaluated using the natural log transform.

In humans, alcohol is converted to acetaldehyde by alcohol dehydrogenases (*ADH)*, and then to acetate by acetaldehyde dehydrogenases (*ALDH)* [45]. Variants in genes from the *ADH* and *ALDH* gene families are often associated with reduced drinking as they raise the blood level of acetaldehyde which causes uncomfortable symptoms such as "hangover", nausea, and facial flush [46]. Therefore, genetic variation in the *ADH* and *ALDH* genes were the focus of this study. Previously published association studies, most of which involved European ancestry populations, identified 18 single nucleotide polymorphisms (SNPs) in *ADH* and *ALDH* genes (S1 Table) that were significantly associated with alcohol consumption [47–61]. These SNPs were evaluated for instrument selection by first identifying their availability in ARIC based on genotypes from the Infinium HumanExome BeadChip v1.0 (Illumina, Inc., San Diego, CA) [62] referred to as the “exome chip” hereafter, genotypes from the Affymetrix 6.0 array [63], or those that were genotyped by TaqMan [64]. From ten SNPs available in ARIC, 6 SNPs in the *ADH* and *ALDH* genes passed stringent quality control procedures [62] (S1 Table).

Next, the 6 SNPs were evaluated for violation of IV assumptions. Since these assumptions are violated if the genetic instruments are in high linkage disequilibrium (LD) with loci associated with lipid levels [19, 23], the SNPs were examined for LD (r^2^ > 0.2) with lipid-related loci identified in published genome wide association studies (GWAS) [65–76]. The SNPs were then evaluated using Pearson's correlation coefficient for association (r > 0.1) with potentially genetically determined confounders of the alcohol consumption-lipid relationship. These confounders included smoking, body mass index (BMI), waist-to-hip ratio, and diabetes. To avoid redundancy, the selected SNPs were also evaluated for pair-wise LD (r^2^ > 0.7), and only the SNP with the most functional impact (i.e., exonic, splicing), followed by the largest sample size, was kept. None of the 6 SNPs were correlated with potential confounders, nor were they in high LD with lipid-related loci, but rs1693482 was removed due to LD with another instrumental SNP (r^2^ = 0.96) (S1 Table). A total of 5 SNPs (rs2066702, rs1693457, rs1789891, rs698, and rs1126671) met the IV assumptions (S1 Table).

The final genetic instruments were coded to ensure consistent effect direction of increasing alcohol consumption [77] and then were combined into an unweighted genetic risk score. The genetic risk score was further evaluated for correlation with lipid-related loci and potential confounders using the same criteria applied for the aforementioned SNP selection. The genetic risk score did not violate these MR assumptions, and was used to fit the IV regression models.

First we evaluated the regression between observed alcohol consumption categories and lipid levels to evaluate the linearity of the association. Two Stage Least Square (2SLS) IV regression, performed in Stata 12 [25], was used for causal inference and estimation of the causal effect size of alcohol consumption on lipid measures. Non-linear relationships between alcohol consumption and lipids were evaluated in the second stage of 2SLS using the predicted alcohol consumption categorized into quartiles and then fitted into the models with lipids. The significance of alcohol consumption was evaluated by both Wald p-values from the test comparing each quartile versus the first quartile and Wald p-values for overall significance of alcohol consumption. Population stratification was controlled for [23] using the first two genetic principal component scores calculated in Eigenstrat [78] based on genome-wide autosomal SNPs from the exome chip array. The IV regression models also controlled for sex and age to increase precision and reduce weak instrument bias [20]. With sdLDL-c and apoB measured at visit 4, alcohol consumption and age at visit 4 were included in the model. Similarly, with all other lipid outcomes measured at baseline, alcohol consumption and age at baseline were used. The IV regression diagnostics included a test of weak instrument bias using the F-value of the first stage regression, and an F-value greater than 10 was considered unbiased [20,21]. Because the effect of alcohol may have a J-shaped relationship with lipids and heavy drinking can be harmful [10, 12,79,80] sensitivity analyses excluding heavy drinkers were conducted to examine the effect of alcohol consumption within the low-to-moderate range. In addition, the relationship between alcohol consumption and lipids can be confounded or biased by reverse causation when individuals with adverse health conditions abstain from drinking. Although the MR approach is known to reduce the problems of confounding and reverse causation in observational data [19–27,81], we also conducted a sensitivity analysis excluding never drinkers, never and heavy drinkers, and never and former drinkers in order to further reduce the potential confounding or reverse causation.

## Results

Table 1 shows the characteristics of the ARIC EA individuals included in this study. Regular alcohol consumption was common, and 43.1% of individuals had one or more drink per week. Low-to-moderate current drinkers accounted for 34.4% and heavy drinkers only accounted for 8.8% of individuals.

**Table 1:** Characteristics of 10,893 ARIC EAs.

Characteristics	n (%) or mean (SD)
Female	5,784 (53.10%)
Age (years)	54.3 (5.7)
Alcohol consumption	
grams/week	45.7 (93.9)
Never Drinkers	1,965 (18.10%)
Former Drinkers	1,848 (17.00%)
Infrequent Drinkers*	2,369 (21.80%)
Low-to-Moderate Current Drinkers*	3,734 (34.36%)
Heavy Current Drinkers*	951 (8.75%)
TG (mg/dL)	137.1 (90.7)
Total cholesterol (mg/dL)	214.5 (40.0)
HDL-c (mg/dL)	50.5 (16.8)
HDL2-c (mg/dL)	13.7 (8.6)
HDL3-c (mg/dL)	36.8 (10.9)
LDL-c (mg/dL)	137.6 (37.6)
sdLDL-c (mg/dL)^†^	45.2 (21.0)
apoB (mg/dL)^†^	100.6 (25.1)
Lp(a) (mg/dL)	8.2 (3.0–19.6) ^¥^
Smoking	
never smokers	4,386 (40.30%)
former smokers	3,851 (35.40%)
current smokers	2,649 (24.30%)
Body mass index (kg/m^2^)	27.0 (4.9)
Waist-to-hip ratio	0.9 (0.1)
Diabetic	955 (8.78%)

SD: standard deviation;

*Infrequent: < 1 drink/wk, Low-to-moderate: ≥ 1 drink & ≤ 210 g/wk for men and ≤105 g/wk for women, Heavy: ≥ 1 drink & > 210 g/wk for men and > 105 g/wk for women;

^†^measured at visit 4, lower sample sizes: N = 8,694 and 8,221 for sdLDL-c and apoB, respectively;

^¥^ median and interquartile range.

The regression between observed alcohol consumption categories and lipid levels (Table 2) shows that increased alcohol consumption was associated with lower LDL-c and higher HDL-c, HDL2-c, and HDL3-c levels. A non-linear trend was observed for TG, total cholesterol, and sdLDL-c, and no significant association was observed with apoB and Lp(a). As a result of this observed non-linear relationship between alcohol consumption and the lipids evaluated as a part of this study, in the second stage of 2SLS the predicted alcohol consumption was categorized into quartiles and then fitted into the models with lipids.

**Table 2:** Association between observed alcohol consumption category and lipids.

Lipids	Alcohol consumption categories	beta	95% CI		p^a^
TG ^¥^	Never Drinkers*	0.00			
	Former/ Infrequent Drinkers	-0.08	-0.11	-0.05	**<0.001**
	Low-to-Moderate Current Drinkers	-0.16	-0.19	-0.13	**<0.001**
	Heavy Current Drinkers	-0.13	-0.17	-0.09	**<0.001**
Total cholesterol	Never Drinkers*	0.00			
	Former/ Infrequent Drinkers	-2.75	-4.99	-0.51	**0.02**
	Low-to-Moderate Current Drinkers	-0.73	-3.03	1.57	0.53
	Heavy Current Drinkers	4.05	0.73	7.37	**0.02**
HDL-c ^¥^	Never Drinkers*	0.00			
	Former/ Infrequent Drinkers	0.03	0.01	0.04	**0.001**
	Low-to-Moderate Current Drinkers	0.14	0.12	0.15	**<0.001**
	Heavy Current Drinkers	0.26	0.23	0.28	**<0.001**
HDL2-c ^¥^	Never Drinkers*	0.00			
	Former/ Infrequent Drinkers	0.07	0.04	0.10	**<0.001**
	Low-to-Moderate Current Drinkers	0.17	0.14	0.20	**<0.001**
	Heavy Current Drinkers	0.30	0.26	0.35	**<0.001**
HDL3-c	Never Drinkers*	0.00			
	Former/ Infrequent Drinkers	0.40	-0.14	0.94	0.15
	Low-to-Moderate Current Drinkers	4.16	3.60	4.73	**<0.001**
	Heavy Current Drinkers	8.70	7.84	9.55	**<0.001**
LDL-c	Never Drinkers*	0.00			
	Former/ Infrequent Drinkers	-2.59	-4.69	-0.49	**0.02**
	Low-to-Moderate Current Drinkers	-4.38	-6.53	-2.23	**<0.001**
	Heavy Current Drinkers	-7.48	-10.70	-4.25	**<0.001**
sdLDL-c ^¥^^#^	Never Drinkers*	0.00			
	Former/ Infrequent Drinkers	-0.04	-0.07	-0.01	**0.02**
	Low-to-Moderate Current Drinkers	-0.05	-0.08	-0.01	**0.01**
	Heavy Current Drinkers	0.01	-0.04	0.06	0.66
apoB ^¥^^#^	Never Drinkers*	0.00			
	Former/ Infrequent Drinkers	-0.01	-0.02	0.01	0.52
	Low-to-Moderate Current Drinkers	-0.01	-0.03	0.01	0.54
	Heavy Current Drinkers	-0.02	-0.04	0.01	0.21
Lp(a) ^¥^	Never Drinkers*	0.00			
	Former/ Infrequent Drinkers	-0.03	-0.10	0.03	0.34
	Low-to-Moderate Current Drinkers	0.01	-0.06	0.08	0.72
	Heavy Current Drinkers	-0.06	-0.16	0.03	0.19

*reference group,

^¥^ ln transformed,

^#^ measured at visit 4,

^a^Wald p-value comparing each alcohol consumption category with never drinkers.

Table 3 shows the 5 instrumental SNPs included in this MR study. The effect direction for alcohol consumption of all these 5 final instrumental SNPs was consistent with previous studies [48,52,55,58–60].

**Table 3:** Final instrumental SNPs.

Genes	SNPs	Minor allele frequency (MAF)	Alcohol consumption-raising allele	Effect on alcohol consumption (β)*
*ADH1B*	rs2066702	0.001	G	0.33
*ADH1B*	rs1693457	0.172	T	0.06
*ADH1B/1C*	rs1789891	0.169	A^†^	0.07
*ADH1C*	rs698	0.409	C^†^	0.05
*ADH4*	rs1126671	0.313	A^†^	0.07

* from a linear model, regressing each individual SNP on alcohol consumption,

^†^ minor allele.

The unweighted score created from the final five instrumental SNPs was associated with increased alcohol consumption at baseline (β = 0.06, p < 0.001) (S2 Table). This score explained approximately 0.1% variance of alcohol consumption (Table 4). Almost all 2SLS models using this score had first-stage F-values greater than 10, suggesting no weak instrument bias [20,21] except for sdLDL-c and apoB that were measured at visit 4 and therefore had reduced sample sizes (Table 4).

**Table 4:** Instrumental Variable analysis using 2SLS.

Lipids	N	Predicted alcohol consumption quartiles†	β*	95% CI		p^a^	p overall^b^	1^st^-stage partial R^2^	1^st^-stage F-value
TG ^¥^	9,911	q1	0.00				**<0.001**	0.12%	12.17
		q2	-0.06	-0.09	-0.02	**0.001**			
		q3	-0.13	-0.20	-0.07	**<0.001**			
		q4	-0.08	-0.17	0.00	**0.049**			
Total cholesterol	9,751	q1	0.00				**<0.001**	0.11%	10.86
		q2	-5.54	-8.23	-2.85	**<0.001**			
		q3	-7.71	-13.26	-2.15	**0.007**			
		q4	-4.56	-11.36	2.25	0.189			
HDL-c ^¥^	10,132	q1	0.00				0.117	0.13%	13.48
		q2	0.01	-0.01	0.03	0.435			
		q3	0.04	0.00	0.07	0.070			
		q4	0.03	-0.02	0.07	0.293			
HDL2-c ^¥^	10,120	q1	0.00				**<0.001**	0.13%	13.48
		q2	0.04	0.00	0.07	**0.040**			
		q3	0.10	0.03	0.17	**0.007**			
		q4	0.06	-0.03	0.15	0.179			
HDL3-c	10,120	q1	0.00				0.916	0.13%	13.48
		q2	-0.19	-0.87	0.49	0.580			
		q3	0.08	-1.23	1.38	0.908			
		q4	0.11	-1.51	1.74	0.892			
LDL-c	9,751	q1	0.00				**<0.001**	0.11%	10.86
		q2	-4.60	-7.18	-2.03	**<0.001**			
		q3	-6.87	-12.24	-1.50	**0.012**			
		q4	-4.57	-11.11	1.96	0.170			
sdLDL-c ^¥^^#^	8,102	q1	0.00				0.054	0.07%	6.00
		q2	-0.04	-0.08	-0.01	**0.014**			
		q3	-0.08	-0.15	-0.005	**0.037**			
		q4	-0.08	-0.17	0.01	0.067			
apoB ^¥^^#^	7,663	q1	0.00				**0.022**	0.08%	6.13
		q2	-0.03	-0.04	-0.01	**0.005**			
		q3	-0.04	-0.07	0.001	0.058			
		q4	-0.04	-0.08	0.01	0.132			
Lp(a) ^¥^	9,924	q1	0.00				0.578	0.14%	13.87
		q2	-0.02	-0.09	0.06	0.657			
		q3	-0.05	-0.20	0.10	0.500			
		q4	-0.01	-0.20	0.18	0.890			

^†^Quartile 1: 1.49–3.63 g/wk, quartile 2: 3.63–4.66 g/wk, quartile 3: 4.66–10.57 g/wk, and quartile 4: 10.57–19.54 g/wk,

*second stage regression coefficient between lipid measures and predicted alcohol consumption quartiles with quartile 1 as the reference group,

^a^Wald p-value comparing each quartile with the quartile 1,

^b^Wald p-value for overall effect of alcohol consumption,

^¥^ ln transformed,

^#^ measured at visit 4

Table 4 shows significant causal relationships between alcohol consumption and TG, total cholesterol, HDL2-c, LDL-c, sdLDL-c, and apoB. Alcohol consumption increased HDL2-c and reduced TG, total cholesterol, LDL-c, sdLDL-c, and apoB levels. For all these lipids, the same non-linear trend was observed and the effect peaked at the third quartile and then reduced or stayed the same (only with sdLDL-c) at the fourth quartile. Compared to the first quartile, the third quartile of alcohol consumption had a 12.3% lower level of TG (β in log scale (β_log_) = -0.13, 95%CI: -0.20, -0.07, p < 0.001), a 7.71 mg/dL lower level of total cholesterol (β = -7.71, 95%CI: -13.26, -2.15, p = 0.007), a 10.3% higher level of HDL2-c (β_log_ = 0.10, 95%CI: 0.03, 0.17, p = 0.007), a 6.87 mg/dL lower level of LDL-c (β = -6.87, 95%CI: -12.24, -1.50, p = 0.012), a 7.4% lower level of sdLDL-c (β_log_ = -0.08, 95%CI: -0.15, -0.005, p = 0.037), and a 3.5% lower level of apoB (β_log_ = -0.04, 95%CI: -0.07, 0.001, p = 0.058). With apoB, the second quartile had a slightly lower effect than the third quartile but it was significant (p = 0.005) due to a narrower 95%CI; and the test of overall effect of alcohol consumption was also significant (p_overall_ = 0.022).

The sensitivity analysis excluding heavy drinkers resulted in similar conclusions (Table 5). Specifically, TG, total cholesterol, HDL2-c, LDL-c, sdLDL-c, and apoB were still significant with similar patterns of effect. The lower F-values observed in Table 5 resulted from a smaller sample size due to the exclusion of the heavy drinkers.

**Table 5:** Sensitivity IV analysis excluding heavy drinkers.

Lipids	N	Predicted alcohol consumption quartiles	β*	95% CI		p^a^	p overall^b^	1^st^-stage partial R^2^	1^st^-stage F-value
TG ^¥^	9,024	q1	0.00				**<0.001**	0.10%	9.41
		q2	-0.06	-0.10	-0.03	**<0.001**			
		q3	-0.15	-0.22	-0.08	**<0.001**			
		q4	-0.11	-0.19	-0.02	**0.018**			
Total cholesterol	8,877	q1	0.00				**<0.001**	0.10%	8.64
		q2	-5.42	-8.21	-2.63	**<0.001**			
		q3	-6.43	-12.45	-0.40	**0.037**			
		q4	-2.78	-10.07	4.51	0.454			
HDL-c ^¥^	9,228	q1	0.00				0.164	0.11%	10.39
		q2	0.01	-0.01	0.03	0.236			
		q3	0.04	0.00	0.07	0.079			
		q4	0.03	-0.02	0.08	0.252			
HDL2-c ^¥^	9,216	q1	0.00				**0.002**	0.11%	10.38
		q2	0.04	0.00	0.07	**0.034**			
		q3	0.09	0.01	0.16	**0.023**			
		q4	0.05	-0.04	0.14	0.277			
HDL3-c	9,216	q1	0.00				0.963	0.11%	10.38
		q2	-0.01	-0.69	0.68	0.989			
		q3	0.23	-1.11	1.56	0.737			
		q4	0.37	-1.29	2.03	0.662			
LDL-c	8,877	q1	0.00				**<0.001**	0.10%	8.64
		q2	-4.45	-7.10	-1.80	**0.001**			
		q3	-4.95	-10.72	0.82	0.093			
		q4	-2.29	-9.24	4.66	0.519			
sdLDL-c ^¥^^#^	7,517	q1	0.00				0.098	0.07%	5.08
		q2	-0.04	-0.07	0.00	**0.049**			
		q3	-0.08	-0.16	-0.01	**0.031**			
		q4	-0.09	-0.18	0.00	0.060			
apoB ^¥^^#^	7,110	q1	0.00				**0.045**	0.07%	5.17
		q2	-0.02	-0.04	0.00	**0.013**			
		q3	-0.04	-0.07	0.00	0.064			
		q4	-0.03	-0.08	0.01	0.152			
Lp(a) ^¥^	9,040	q1	0.00				0.742	0.12%	10.43
		q2	-0.02	-0.10	0.06	0.657			
		q3	-0.05	-0.21	0.10	0.502			
		q4	-0.03	-0.23	0.17	0.782			

*second stage regression coefficient between lipid measures and predicted alcohol consumption quartiles with quartile 1 as the reference group,

^a^Wald p-value comparing each quartile with the quartile 1,

^b^Wald p-value for overall effect of alcohol consumption,

^¥^ ln transformed,

^#^ measured at visit 4

The sensitivity analyses excluding never drinkers, never and heavy drinkers, and never and former drinkers (S3–S5 Tables) further confirms the significant causal role of alcohol consumptions on TG, total cholesterol, HDL2-c, LDL-c, sdLDL-c, and apoB.

## Discussion

This study supports the causal role of regular low-to-moderate alcohol consumption in increasing HDL2-c, and reducing TG, total cholesterol and LDL-c, and provides evidence for the novel finding that low-to-moderate consumption of alcohol reduces apoB and sdLDL-c levels among EA. The association between alcohol consumption and increased HDL2-c levels was found in several observational studies [7,82]. A previous MR study also supported a causal effect of alcohol consumption in reducing TG [28]. The relationship between alcohol use and reduction in LDL-c is also concordant with two experimental studies involving red wine [14,15].

The IV analyses conducted in this study demonstrate a non-linear effect of alcohol consumption on TG, total cholesterol, HDL2-c, LDL-c, LDL-c, sdLDL-c, and apoB, with the highest effects observed at the third quartile of alcohol consumption. This suggests that alcohol consumption may have the greatest benefit within a low-to-moderate range. When excluding heavy drinkers, the effect of alcohol consumption on those lipids remained significant, and with the peak effect at the third quartile. Therefore we conclude that alcohol consumption may have the greatest benefit within a low-to-moderate range and higher drinking does not always result in larger benefit. The consistent results when excluding never and former drinkers further confirm that the causal role of alcohol consumption on TG, total cholesterol, HDL2-c, LDL-c, LDL-c, sdLDL-c, and apoB are robust to potential confounding and reverse causation from adverse health conditions that may prevent people from drinking.

These findings may help explain the mechanism of a cardiovascular protective effect of alcohol consumption. Studies found HDL2-c, which are large HDL particles, had cardioprotective effect [83–85]. Alcohol consumption may raise HDL-c level by increasing hepatic production or increasing transport rate of apoA-I and apoA-II [7,86,87], increasing cellular cholesterol efflux and plasma cholesterol esterification [7,87,88], increasing muscle ATP-binding cassette, subfamily A (ABCA1) which may be important in recycling preformed HDL through reverse cholesterol transport, and decreasing cholesteryl ester transfer protein (CETP) [7,87]. Lowered CETP level was found associated with an increased level of large HDL particles [89]. sdLDL is considered a pro-atherogenic particle due to their susceptibility to oxidization that promotes inflammation and plaque development [44,90–92]. Studies suggest that apoB may be more predictive than LDL-c for the risk of CHD [91,93,94], and total apoB likely reflect the total number of atherogenic particles [92].

This study has a number of strengths. With the large sample size, this is one of the most comprehensive MR studies involving alcohol use and a comprehensive set of lipid measures. This is also the first MR study looking at the causal effect of alcohol consumption on HDL-c subfractions, sdLDL-c, apoB, and Lp(a). Compared to a case-control study, the cohort design helps to avoid selection bias and increases the validity of the MR approach [19]. This study employed a stringent process of selecting genetic instruments. The instrumental variants were examined by a thorough procedure of validating MR assumptions and possible violations including linkage disequilibrium (LD) and pleiotropy issues. The potential for population stratification was also addressed by controlling for genetic principal components in the IV regression models. The fact that the score was created from different genes further strengthens the causal inference in this study, because potential violations of MR assumptions through LD and pleiotropy issues were unlikely [23].

A limitation of this study is the fact that sdLDL-c and apoB were measured at visit 4 and the sample sizes were reduced compared to the analyses of other lipid measures at baseline. This resulted in F-values less than 10, indicating that the results have the potential for bias, and therefore the causal inference for sdLDL-c and apoB should be interpreted with caution. Several instrumental SNPs were related to alcohol dependence and alcoholism in previous studies. Given that the variants of interest are located in the *ADH* genes, the likely biological mechanism of the mutated alleles is to increase acetaldehyde level and prevent people from drinking. Therefore, those *ADH* genetic variants should influence alcohol consumption level, not just the status of dependence. This was confirmed by significant associations between the genetic score and alcohol consumption at baseline as well as at visit 4 (S2 Table).

In conclusion, this study supports the role of low-to-moderate alcohol use in improving lipid profiles. Continued investigation of the role of alcohol consumption on TG, total cholesterol, HDL2-c, LDL-c, sdLDL-c, and apoB is warranted.

## Supporting Information

S1 Table Genetic instrument selection (DOCX)

S2 Table Association between unweighted genetic score and alcohol consumption (DOCX)

S3 Table Sensitivity IV analysis excluding never drinkers (DOCX)

S4 Table Sensitivity IV analysis excluding never and heavy drinkers (DOCX)

S5 Table Sensitivity IV analysis excluding never and former drinkers (DOCX)

## References

[1] Ronksley PE, Brien SE, Turner BJ, Mukamal KJ, Ghali WA. Association of alcohol consumption with selected cardiovascular disease outcomes: a systematic review and meta-analysis. BMJ. 2011;342:d671 10.1136/bmj.d671 21343207 PMC3043109

[2] Han H, Wang H, Yin Z, Jiang H, Fang M, Han J. Association of genetic polymorphisms in ADH and ALDH2 with risk of coronary artery disease and myocardial infarction: a meta-analysis. Gene. 2013;526(2):134–41. 10.1016/j.gene.2013.05.002 23685282

[3] Hines LM, Stampfer MJ, Ma J, Gaziano JM, Ridker PM, Hankinson SE, et al Genetic variation in alcohol dehydrogenase and the beneficial effect of moderate alcohol consumption on myocardial infarction. N Engl J Med. 2001;344(8):549–55. 11207350 10.1056/NEJM200102223440802

[4] Brien SE, Ronksley PE, Turner BJ, Mukamal KJ, Ghali WA. Effect of alcohol consumption on biological markers associated with risk of coronary heart disease: systematic review and meta-analysis of interventional studies. BMJ. 2011;342:d636 10.1136/bmj.d636 21343206 PMC3043110

[5] Rimm EB, Williams P, Fosher K, Criqui M, Stampfer MJ. Moderate alcohol intake and lower risk of coronary heart disease: meta-analysis of effects on lipids and haemostatic factors. BMJ. 1999;319(7224):1523–8. 10591709 10.1136/bmj.319.7224.1523 PMC28294

[6] Gardner CD, Tribble DL, Young DR, Ahn D, Fortmann SP. Associations of HDL, HDL(2), and HDL(3) cholesterol and apolipoproteins A-I and B with lifestyle factors in healthy women and men: the Stanford Five City Project. Prev Med. 2000;31(4):346–56. 11006059 10.1006/pmed.2000.0716

[7] Muth ND, Laughlin GA, von Muhlen D, Smith SC, Barrett-Connor E. High-density lipoprotein subclasses are a potential intermediary between alcohol intake and reduced risk of cardiovascular disease: the Rancho Bernardo Study. Br J Nutr. 2010;104(7):1034–42. 10.1017/S0007114510001595 20426890

[8] Wakabayashi I. Associations of alcohol drinking and cigarette smoking with serum lipid levels in healthy middle-aged men. Alcohol Alcohol. 2008;43(3):274–80. 10.1093/alcalc/agn005 18283096

[9] Rakic V, Puddey IB, Dimmitt SB, Burke V, Beilin LJ. A controlled trial of the effects of pattern of alcohol intake on serum lipid levels in regular drinkers. Atherosclerosis. 1998;137(2):243–52. 9622267 10.1016/s0021-9150(97)00269-4

[10] Wakabayashi I. Relationship between alcohol intake and lipid accumulation product in middle-aged men. Alcohol Alcohol. 2013;48(5):535–42. 10.1093/alcalc/agt032 23592501

[11] Klop B, do Rego AT, Cabezas MC. Alcohol and plasma triglycerides. Curr Opin Lipidol. 2013;24(4):321–6. 10.1097/MOL.0b013e3283606845 23511381

[12] Whitfield JB, Heath AC, Madden PA, Pergadia ML, Montgomery GW, Martin NG. Metabolic and biochemical effects of low-to-moderate alcohol consumption. Alcohol Clin Exp Res. 2013;37(4):575–86. 10.1111/acer.12015 23134229 PMC3568441

[13] Brinton EA. Effects of ethanol intake on lipoproteins. Current atherosclerosis reports. 2012;14(2):108–14. 10.1007/s11883-012-0230-7 22350634

[14] Kechagias S, Zanjani S, Gjellan S, Leinhard OD, Kihlberg J, Smedby O, et al Effects of moderate red wine consumption on liver fat and blood lipids: a prospective randomized study. Ann Med. 2011;43(7):545–54. 10.3109/07853890.2011.588246 21599573

[15] Rifler JP, Lorcerie F, Durand P, Delmas D, Ragot K, Limagne E, et al A moderate red wine intake improves blood lipid parameters and erythrocytes membrane fluidity in post myocardial infarct patients. Mol Nutr Food Res. 2012;56(2):345–51. 10.1002/mnfr.760 22419533

[16] Volcik KA, Ballantyne CM, Fuchs FD, Sharrett AR, Boerwinkle E. Relationship of alcohol consumption and type of alcoholic beverage consumed with plasma lipid levels: differences between Whites and African Americans of the ARIC study. Ann Epidemiol. 2008;18(2):101–7. 17855114 10.1016/j.annepidem.2007.07.103 PMC2819069

[17] Naimi TS, Brown DW, Brewer RD, Giles WH, Mensah G, Serdula MK, et al Cardiovascular risk factors and confounders among nondrinking and moderate-drinking U.S. adults. Am J Prev Med. 2005;28(4):369–73. 15831343 10.1016/j.amepre.2005.01.011

[18] Mukamal KJ, Ding EL, Djousse L. Alcohol consumption, physical activity, and chronic disease risk factors: a population-based cross-sectional survey. BMC Public Health. 2006;6:118 16670030 10.1186/1471-2458-6-118 PMC1475847

[19] Didelez V, Sheehan N. Mendelian randomization as an instrumental variable approach to causal inference. Stat Methods Med Res. 2007;16(4):309–30. 17715159 10.1177/0962280206077743

[20] Burgess S, Thompson SG, Collaboration CCG. Avoiding bias from weak instruments in Mendelian randomization studies. Int J Epidemiol. 2011;40(3):755–64. 10.1093/ije/dyr036 21414999

[21] Pierce BL, Ahsan H, Vanderweele TJ. Power and instrument strength requirements for Mendelian randomization studies using multiple genetic variants. Int J Epidemiol. 2011;40(3):740–52. 10.1093/ije/dyq151 20813862 PMC3147064

[22] Murray MP. Avoiding invalid instruments and coping with weak instruments. The Journal of Economic Perspectives. 2006;20(4):111–32.

[23] Thanassoulis G. Mendelian randomization: how genetics is pushing the boundaries of epidemiology to identify new causes of heart disease. Can J Cardiol. 2013;29(1):30–6. 10.1016/j.cjca.2012.09.014 23199790

[24] Rassen JA, Schneeweiss S, Glynn RJ, Mittleman MA, Brookhart MA. Instrumental variable analysis for estimation of treatment effects with dichotomous outcomes. Am J Epidemiol. 2009;169(3):273–84. 10.1093/aje/kwn299 19033525

[25] Baum CF, Schaffer ME, Stillman S. Enhanced routines for instrumental variables/GMM estimation and testing. Stata Journal. 2007;7(4):465–506.

[26] Baum C. Instrumental variables and panel data methods in economics and finance. Boston College and DIW Berlin 2009.

[27] Burgess S, Thompson SG. Use of allele scores as instrumental variables for Mendelian randomization. Int J Epidemiol. 2013;42(4):1134–44. 10.1093/ije/dyt093 24062299 PMC3780999

[28] Lawlor DA, Nordestgaard BG, Benn M, Zuccolo L, Tybjaerg-Hansen A, Davey Smith G. Exploring causal associations between alcohol and coronary heart disease risk factors: findings from a Mendelian randomization study in the Copenhagen General Population Study. Eur Heart J. 2013;34(32):2519–28. 10.1093/eurheartj/eht081 23492672

[29] Au Yeung SL, Jiang C, Cheng KK, Cowling BJ, Liu B, Zhang W, et al Moderate alcohol use and cardiovascular disease from Mendelian randomization. PLOS One. 2013;8(7):e68054 10.1371/journal.pone.0068054 23874492 PMC3712994

[30] Martin SS, Jones SR, Toth PP. High-density lipoprotein subfractions: current views and clinical practice applications. Trends Endocrinol Metab. 2014;25(7):329–36. 10.1016/j.tem.2014.05.005 24931711

[31] The ARIC Investigators. The Atherosclerosis Risk in Communities (ARIC) Study: design and objectives. Am J Epidemiol. 1989;129(4):687–702. 2646917

[32] U.S. Department of Agriculture and U.S. Department of Health and Human Services. Dietary Guidelines for Americans, 2010. 7th ed Washington, DC: U.S. Government Printing Office; 12 2010. 10.3945/an.111.000430 PMC3090168 22332062

[33] Agrawal A, Freedman ND, Cheng YC, Lin P, Shaffer JR, Sun Q, et al Measuring alcohol consumption for genomic meta-analyses of alcohol intake: opportunities and challenges. Am J Clin Nutr. 2012;95(3):539–47. 10.3945/ajcn.111.015545 22301922 PMC3278237

[34] Chobanian AV, Bakris GL, Black HR, Cushman WC, Green LA, Izzo JL Jr, et al The seventh report of the joint national committee on prevention, detection, evaluation, and treatment of high blood pressure: the JNC 7 report. JAMA. 2003;289(19):2560–71. 12748199 10.1001/jama.289.19.2560

[35] Wakabayashi I, Kobaba-Wakabayashi R, Masuda H. Relation of drinking alcohol to atherosclerotic risk in type 2 diabetes. Diabetes Care. 2002;25(7):1223–8. 12087023 10.2337/diacare.25.7.1223

[36] Siedel J, Hagele EO, Ziegenhorn J, Wahlefeld AW. Reagent for the enzymatic determination of serum total cholesterol with improved lipolytic efficiency. Clin Chem. 1983;29(6):1075–80. 6851096

[37] Nagele U, Hagele EO, Sauer G, Wiedemann E, Lehmann P, Wahlefeld AW, et al Reagent for the enzymatic determination of serum total triglycerides with improved lipolytic efficiency. J Clin Chem Clin Biochem. 1984;22(2):165–74. 6716056 10.1515/cclm.1984.22.2.165

[38] Warnick GR, Mayfield C, Benderson J, Chen JS, Albers JJ. HDL cholesterol quantitation by phosphotungstate-Mg2+ and by dextran sulfate-Mn2+-polyethylene glycol precipitation, both with enzymic cholesterol assay compared with the lipid research method. Am J Clin Pathol. 1982;78(5):718–23. 6182791 10.1093/ajcp/78.5.718

[39] Brown SA, Hutchinson R, Morrisett J, Boerwinkle E, Davis CE, Gotto AM Jr., et al Plasma lipid, lipoprotein cholesterol, and apoprotein distributions in selected US communities. The Atherosclerosis Risk in Communities (ARIC) Study. Arterioscler Thromb. 1993;13(8):1139–58. 8343489 10.1161/01.atv.13.8.1139

[40] Friedewald WT, Levy RI, Fredrickson DS. Estimation of the concentration of low-density lipoprotein cholesterol in plasma, without use of the preparative ultracentrifuge. Clin Chem. 1972;18(6):499–502. 4337382

[41] Shahar E, Chambless LE, Rosamond WD, Boland LL, Ballantyne CM, McGovern PG, et al Plasma lipid profile and incident ischemic stroke: the Atherosclerosis Risk in Communities (ARIC) study. Stroke. 2003;34(3):623–31. 12624282 10.1161/01.STR.0000057812.51734.FF

[42] Gaubatz JW, Chari MV, Nava ML, Guyton JR, Morrisett JD. Isolation and characterization of the two major apoproteins in human lipoprotein [a]. J Lipid Res. 1987;28(1):69–79. 2951469

[43] Virani SS, Brautbar A, Davis BC, Nambi V, Hoogeveen RC, Sharrett AR, et al Associations between lipoprotein(a) levels and cardiovascular outcomes in black and white subjects: the Atherosclerosis Risk in Communities (ARIC) Study. Circulation. 2012;125(2):241–9. 10.1161/CIRCULATIONAHA.111.045120 22128224 PMC3760720

[44] Hoogeveen RC, Gaubatz JW, Sun W, Dodge RC, Crosby JR, Jiang J, et al Small dense low-density lipoprotein-cholesterol concentrations predict risk for coronary heart disease: the Atherosclerosis Risk In Communities (ARIC) study. Arterioscler Thromb Vasc Biol. 2014;34(5):1069–77. 10.1161/ATVBAHA.114.303284 24558110 PMC3999643

[45] Zakhari S. Overview: how is alcohol metabolized by the body? Alcohol Res Health. 2006;29(4):245–54. 17718403 PMC6527027

[46] Hurley TD, Edenberg HJ. Genes encoding enzymes involved in ethanol metabolism. Alcohol Res. 2012;34(3):339–44. 23134050 10.35946/arcr.v34.3.09 PMC3756590

[47] Rietschel M, Treutlein J. The genetics of alcohol dependence. Ann N Y Acad Sci. 2013;1282:39–70. 10.1111/j.1749-6632.2012.06794.x 23170934

[48] Gelernter J, Kranzler HR, Sherva R, Almasy L, Koesterer R, Smith AH, et al Genome-wide association study of alcohol dependence:significant findings in African- and European-Americans including novel risk loci. Mol Psychiatry. 2014;19(1):41–9. 10.1038/mp.2013.145 24166409 PMC4165335

[49] Macgregor S, Lind PA, Bucholz KK, Hansell NK, Madden PA, Richter MM, et al Associations of ADH and ALDH2 gene variation with self report alcohol reactions, consumption and dependence: an integrated analysis. Hum Mol Genet. 2009;18(3):580–93. 10.1093/hmg/ddn372 18996923 PMC2722191

[50] Luo X, Kranzler HR, Zuo L, Yang BZ, Lappalainen J, Gelernter J. ADH4 gene variation is associated with alcohol and drug dependence: results from family controlled and population-structured association studies. Pharmacogenet Genomics. 2005;15(11):755–68. 16220108 10.1097/01.fpc.0000180141.77036.dc

[51] Ferrari P, McKay JD, Jenab M, Brennan P, Canzian F, Vogel U, et al Alcohol dehydrogenase and aldehyde dehydrogenase gene polymorphisms, alcohol intake and the risk of colorectal cancer in the European Prospective Investigation into Cancer and Nutrition study. Eur J Clin Nutr. 2012;66(12):1303–8. 10.1038/ejcn.2012.173 23149980

[52] Way M, McQuillin A, Saini J, Ruparelia K, Lydall GJ, Guerrini I, et al Genetic variants in or near ADH1B and ADH1C affect susceptibility to alcohol dependence in a British and Irish population. Addict Biol. 2015;20(3):594–604. 10.1111/adb.12141 24735490

[53] Biernacka JM, Geske JR, Schneekloth TD, Frye MA, Cunningham JM, Choi DS, et al Replication of genome wide association studies of alcohol dependence: support for association with variation in ADH1C. PLOS One. 2013;8(3):e58798 10.1371/journal.pone.0058798 23516558 PMC3596339

[54] Li D, Zhao H, Gelernter J. Strong association of the alcohol dehydrogenase 1B gene (ADH1B) with alcohol dependence and alcohol-induced medical diseases. Biol Psychiatry. 2011;70(6):504–12. 10.1016/j.biopsych.2011.02.024 21497796 PMC3142297

[55] Toth R, Fiatal S, Petrovski B, McKee M, Adany R. Combined effect of ADH1B RS1229984, RS2066702 and ADH1C RS1693482/ RS698 alleles on alcoholism and chronic liver diseases. Dis Markers. 2011;31(5):267–77. 10.3233/DMA-2011-0828 22048268 PMC3826929

[56] Norden-Krichmar TM, Gizer IR, Wilhelmsen KC, Schork NJ, Ehlers CL. Protective variant associated with alcohol dependence in a Mexican American cohort. BMC Med Genet. 2014;15:136 10.1186/s12881-014-0136-z 25527893 PMC4337107

[57] Bierut LJ, Goate AM, Breslau N, Johnson EO, Bertelsen S, Fox L, et al ADH1B is associated with alcohol dependence and alcohol consumption in populations of European and African ancestry. Mol Psychiatry. 2012;17(4):445–50. 10.1038/mp.2011.124 21968928 PMC3252425

[58] Li D, Zhao H, Gelernter J. Further clarification of the contribution of the ADH1C gene to vulnerability of alcoholism and selected liver diseases. Hum Genet. 2012;131(8):1361–74. 10.1007/s00439-012-1163-5 22476623 PMC3557796

[59] Agrawal A, Bierut LJ. Identifying genetic variation for alcohol dependence. Alcohol Res. 2012;34(3):274–81. 23134043 10.35946/arcr.v34.3.02 PMC3662475

[60] Agrawal A, Verweij KJ, Gillespie NA, Heath AC, Lessov-Schlaggar CN, Martin NG, et al The genetics of addiction-a translational perspective. Transl Psychiatry. 2012;2:e140 10.1038/tp.2012.54 22806211 PMC3410620

[61] Zuccolo L, Fitz-Simon N, Gray R, Ring SM, Sayal K, Smith GD, et al A non-synonymous variant in ADH1B is strongly associated with prenatal alcohol use in a European sample of pregnant women. Hum Mol Genet. 2009;18(22):4457–66. 10.1093/hmg/ddp388 19687126 PMC2766294

[62] Grove ML, Yu B, Cochran BJ, Haritunians T, Bis JC, Taylor KD, et al Best practices and joint calling of the HumanExome BeadChip: the CHARGE Consortium. PLOS One. 2013;8(7):e68095 10.1371/journal.pone.0068095 23874508 PMC3709915

[63] Genome-Wide Human SNP Array 6.0. Available: http://www.affymetrix.com/catalog/131533/AFFY/Genome-Wide+Human+SNP+Array+6.0#1_1.

[64] Gene Expression Analysis Using TaqMan^®^ Assays: Life Technologies. Available: http://www.lifetechnologies.com/us/en/home/life-science/pcr/real-time-pcr/real-time-pcr-assays/taqman-gene-expression.html.

[65] Willer CJ, Schmidt EM, Sengupta S, Peloso GM, Gustafsson S, Kanoni S, et al Discovery and refinement of loci associated with lipid levels. Nat Genet. 2013;45(11):1274–83. 10.1038/ng.2797 24097068 PMC3838666

[66] Teslovich TM, Musunuru K, Smith AV, Edmondson AC, Stylianou IM, Koseki M, et al Biological, clinical and population relevance of 95 loci for blood lipids. Nature. 2010;466(7307):707–13. 10.1038/nature09270 20686565 PMC3039276

[67] Adeyemo A, Bentley AR, Meilleur KG, Doumatey AP, Chen G, Zhou J, et al Transferability and fine mapping of genome-wide associated loci for lipids in African Americans. BMC Med Genet. 2012;13:88 10.1186/1471-2350-13-88 22994408 PMC3573912

[68] Rasmussen-Torvik LJ, Pacheco JA, Wilke RA, Thompson WK, Ritchie MD, Kho AN, et al High density GWAS for LDL cholesterol in African Americans using electronic medical records reveals a strong protective variant in APOE. Clin Transl Sci. 2012;5(5):394–9. 10.1111/j.1752-8062.2012.00446.x 23067351 PMC3521536

[69] Keebler ME, Deo RC, Surti A, Konieczkowski D, Guiducci C, Burtt N, et al Fine-mapping in African Americans of 8 recently discovered genetic loci for plasma lipids: the Jackson Heart Study. Circ Cardiovasc Genet. 2010;3(4):358–64. 10.1161/CIRCGENETICS.109.914267 20570916 PMC3074173

[70] Carlson CS, Matise TC, North KE, Haiman CA, Fesinmeyer MD, Buyske S, et al Generalization and dilution of association results from European GWAS in populations of non-European ancestry: the PAGE study. PLOS Biol. 2013;11(9):e1001661 10.1371/journal.pbio.1001661 24068893 PMC3775722

[71] Deo RC, Reich D, Tandon A, Akylbekova E, Patterson N, Waliszewska A, et al Genetic differences between the determinants of lipid profile phenotypes in African and European Americans: the Jackson Heart Study. PLOS genetics. 2009;5(1):e1000342 10.1371/journal.pgen.1000342 19148283 PMC2613537

[72] Dumitrescu L, Carty CL, Taylor K, Schumacher FR, Hindorff LA, Ambite JL, et al Genetic determinants of lipid traits in diverse populations from the population architecture using genomics and epidemiology (PAGE) study. PLOS genetics. 2011;7(6):e1002138 10.1371/journal.pgen.1002138 21738485 PMC3128106

[73] Liu CT, Monda KL, Taylor KC, Lange L, Demerath EW, Palmas W, et al Genome-wide association of body fat distribution in African ancestry populations suggests new loci. PLOS genetics. 2013;9(8):e1003681 10.1371/journal.pgen.1003681 23966867 PMC3744443

[74] Musunuru K, Romaine SP, Lettre G, Wilson JG, Volcik KA, Tsai MY, et al Multi-ethnic analysis of lipid-associated loci: the NHLBI CARe project. PLOS One. 2012;7(5):e36473 10.1371/journal.pone.0036473 22629316 PMC3357427

[75] Bryant EK, Dressen AS, Bunker CH, Hokanson JE, Hamman RF, Kamboh MI, et al A multiethnic replication study of plasma lipoprotein levels-associated SNPs identified in recent GWAS. PLOS One. 2013;8(5):e63469 10.1371/journal.pone.0063469 23717430 PMC3661596

[76] Lettre G, Palmer CD, Young T, Ejebe KG, Allayee H, Benjamin EJ, et al Genome-wide association study of coronary heart disease and its risk factors in 8,090 African Americans: the NHLBI CARe Project. PLOS genetics. 2011;7(2):e1001300 10.1371/journal.pgen.1001300 21347282 PMC3037413

[77] Morrison AC, Bare LA, Chambless LE, Ellis SG, Malloy M, Kane JP, et al Prediction of coronary heart disease risk using a genetic risk score: the Atherosclerosis Risk in Communities Study. Am J Epidemiol. 2007;166(1):28–35. 17443022 10.1093/aje/kwm060

[78] Havard School of Public Health. EIGENSOFT. Available: http://www.hsph.harvard.edu/alkes-price/software/.

[79] Chrysohoou C, Panagiotakos DB, Pitsavos C, Skoumas J, Toutouza M, Papaioannou I, et al Effects of chronic alcohol consumption on lipid levels, inflammatory and haemostatic factors in the general population: the 'ATTICA' Study. Eur J Cardiovasc Prev Rehabil. 2003;10(5):355–61. 14663297 10.1097/01.hjr.0000065928.57001.4d

[80] Park H, Kim K. Association of alcohol consumption with lipid profile in hypertensive men. Alcohol Alcohol. 2012;47(3):282–7. 10.1093/alcalc/ags019 22371847

[81] Foster EM. Instrumental variables for logistic regression: an illustration. Soc Sci Res. 1997;26(4):487–504.

[82] Gaziano JM, Buring JE, Breslow JL, Goldhaber SZ, Rosner B, VanDenburgh M, et al Moderate alcohol intake, increased levels of high-density lipoprotein and its subfractions, and decreased risk of myocardial infarction. N Engl J Med. 1993;329(25):1829–34. 8247033 10.1056/NEJM199312163292501

[83] Superko HR, Pendyala L, Williams PT, Momary KM, King SB 3rd, Garrett BC. High-density lipoprotein subclasses and their relationship to cardiovascular disease. J Clin Lipidol. 2012;6(6):496–523. 10.1016/j.jacl.2012.03.001 23312047

[84] Williams PT. Fifty-three year follow-up of coronary heart disease versus HDL2 and other lipoproteins in Gofman's Livermore Cohort. J Lipid Res. 2012;53(2):266–72. 10.1194/jlr.M019356 22128321 PMC3269162

[85] Williams PT, Feldman DE. Prospective study of coronary heart disease vs. HDL2, HDL3, and other lipoproteins in Gofman's Livermore Cohort. Atherosclerosis. 2011;214(1):196–202. 10.1016/j.atherosclerosis.2010.10.024 21109246 PMC3786414

[86] De Oliveira ESER, Foster D, McGee Harper M, Seidman CE, Smith JD, Breslow JL, et al Alcohol consumption raises HDL cholesterol levels by increasing the transport rate of apolipoproteins A-I and A-II. Circulation. 2000;102(19):2347–52. 11067787 10.1161/01.cir.102.19.2347

[87] Beulens JW, Sierksma A, van Tol A, Fournier N, van Gent T, Paul JL, et al Moderate alcohol consumption increases cholesterol efflux mediated by ABCA1. J Lipid Res. 2004;45(9):1716–23. 15231854 10.1194/jlr.M400109-JLR200

[88] van der Gaag MS, van Tol A, Vermunt SH, Scheek LM, Schaafsma G, Hendriks HF. Alcohol consumption stimulates early steps in reverse cholesterol transport. J Lipid Res. 2001;42(12):2077–83. 11734581

[89] Ordovas JM, Cupples LA, Corella D, Otvos JD, Osgood D, Martinez A, et al Association of cholesteryl ester transfer protein-TaqIB polymorphism with variations in lipoprotein subclasses and coronary heart disease risk: the Framingham study. Arterioscler Thromb Vasc Biol. 2000;20(5):1323–9. 10807749 10.1161/01.atv.20.5.1323

[90] Walldius G, Jungner I. Apolipoprotein B and apolipoprotein A-I: risk indicators of coronary heart disease and targets for lipid-modifying therapy. J Intern Med. 2004;255(2):188–205. 14746556 10.1046/j.1365-2796.2003.01276.x

[91] Brunzell JD. Increased ApoB in small dense LDL particles predicts premature coronary artery disease. Arterioscler Thromb Vasc Biol. 2005;25(3):474–5. 15731485 10.1161/01.ATV.0000156537.78366.1d

[92] Walldius G, Jungner I. Rationale for using apolipoprotein B and apolipoprotein A-I as indicators of cardiac risk and as targets for lipid-lowering therapy. Eur Heart J. 2005;26(3):210–2. 15618031 10.1093/eurheartj/ehi077

[93] Jacobson TA. Opening a new lipid "apo-thecary": incorporating apolipoproteins as potential risk factors and treatment targets to reduce cardiovascular risk. Mayo Clin Proc. 2011;86(8):762–80. 10.4065/mcp.2011.0128 21803958 PMC3146376

[94] Contois JH, Warnick GR, Sniderman AD. Reliability of low-density lipoprotein cholesterol, non-high-density lipoprotein cholesterol, and apolipoprotein B measurement. J Clin Lipidol. 2011;5(4):264–72. 10.1016/j.jacl.2011.05.004 21784371

