# Supplementary material for: Causal Role of Alcohol Consumption in an Improved Lipid Profile: The Atherosclerosis Risk in Communities (ARIC) Study
Source: PLoS One. 2016 Feb 5;11(2):e0148765. doi: 10.1371/journal.pone.0148765 (PMC4744040; doi:10.1371/journal.pone.0148765)
Supplement: S1 Table — (DOCX) [file pone.0148765.s001.docx]

**Supporting information**

**Causal role of alcohol consumption in an improved lipid profile: the Atherosclerosis Risk in Communities (ARIC) study**

Khanh N. Vu^1^, Christie M. Ballantyne^2,3^_,_ Ron C. Hoogeveen^2,3^, Vijay Nambi^2,3,4^, Kelly A. Volcik^5^, Eric Boerwinkle^1,6^ Alanna C. Morrison^1*^

^1^School of Public Health, University of Texas Health Science Center at Houston, Houston, TX, USA

^2^Section of Cardiovascular Research, Baylor College of Medicine, Houston, TX, USA

^3^Houston Methodist Debakey Heart and Vascular Center, Houston, TX, USA

^4^Michael E DeBakey Veterans Affairs Hospital, Houston, TX, USA

^5^Department of Biochemistry and Molecular Biology, University of Texas Medical School at Houston, Houston, TX, USA

^6^The Human Genome Sequencing Center, Baylor College of Medicine, Houston, TX, USA

*Corresponding author

E-mail: Alanna.C.Morrison@uth.tmc.edu (ACM)

**S1 Table. Genetic instrument selection**

| Chr | Genes | rs number | Functional class | A1 | A2 | Effect direction of A1* | References | Available in ARIC | LD with lipid loci (r^2^)† | Correlation with confounders (r)¥ | Final instruments |
| --- | --- | --- | --- | --- | --- | --- | --- | --- | --- | --- | --- |
| 4 | *ADH1A* | rs904092 | upstream | A | G | NA | Gelernter et al, 2014[1] | No |  |  |  |
| 4 | *ADH1A* | rs2866151 | intronic | T | A | + | Zuccolo et al, 2009[2] | No |  |  |  |
| 4 | *ADH1B* | rs1042026 | downstream | G | A | - | Macgregor et al, 2009[3] | Yes |  |  | No, high missing rate |
| 4 | *ADH1B* | rs1229984 | exonic | A | G | - | Gelernter et al, 2014[1]; Zuccolo et al, 2009[2]; Agrawal et al, 2012[4]; Ferrari et al, 2012[5]; Li et al, 2011[6]; Bierut et al, 2012[7]; Way et al, 2015[8] | Yes |  |  | No, did not pass quality control‡ |
| 4 | *ADH1B* | rs2066702 | exonic | A | G | - | Gelernter et al, 2014[1] | Yes | 0.003 | 0.011 | Yes |
| 4 | *ADH1B* | rs1789882 | exonic | A | G | - | Gelernter et al, 2014[1] | No |  |  |  |
| 4 | *ADH1B* | rs1693457 | intronic | C | T | - | Gelernter et al, 2014[1] | Yes | 0.001 | 0.015 | Yes |
| 4 | *ADH1B/1C* | rs1789891 | intergenic | A | C | + | Way et al, 2015[8]; Agrawal et al, 2012[9] | Yes | 0.001 | 0.023 | Yes |
| 4 | *ADH1C* | rs1693482 | exonic | A | G | + | Macgregor et al, 2009[3]; Agrawal et al, 2012[4]; Way et al, 2015[8]; Toth et al, 2011[10] | Yes | 0.000 | 0.011 | No, in high LD with rs698 and has lower sample size |
| 4 | *ADH1C* | rs698 | exonic | C | T | + | Agrawal et al, 2012[4]; Way et al, 2015[8]; Toth et al, 2011[10]; Li et al, 2012[11] | Yes | 0.001 | 0.015 | Yes |
| 4 | *ADH1C* | rs283413 | exonic | T | G | - | Way et al, 2015[8]; Biernacka et al, 2013[12]; Norden-Krichmar et al, 2014[13] | No |  |  |  |
| 4 | *ADH1C* | rs2241894 | exonic | C | T | - | Gelernter et al, 2014[1] | No |  |  |  |
| 4 | *ADH1C* | rs1614972 | intronic | T | C | - | Gelernter et al, 2014[1]; Agrawal et al, 2012[4] | Yes |  |  | No, violation of HWE assumptions |
| 4 | *ADH4* | rs3762894 | upstream | G | A | NA | Macgregor et al, 2009[3] | No |  |  |  |
| 4 | *ADH4* | rs1042363 | exonic | T | C | - | Luo et al, 2005[14] | No |  |  |  |
| 4 | *ADH4* | rs1126671 | exonic | A | G | + | Luo et al, 2005[14] | Yes | 0.001 | 0.014 | Yes |
| 4 | *ADH5* | rs1230165 | downstream | C | T | NA | Macgregor et al, 2009[3] | No |  |  |  |
| 12 | *ALDH2* | rs671 | exonic | A | G | - | Agrawal et al, 2012[4]; Rietschel et al, 2013[15] | Yes |  |  | No, monomorphic |

Chr: chromosome, A1: minor allele, A2: major allele, *effect direction: + minor allele increases consumption, - minor allele decreases consumption, NA: not available, † highest LD with lipid loci, ¥ highest correlation with confounders, ‡ low call rate.

Of the ten SNPs available in ARIC data, five were excluded due to the following reasons: monomorphism (rs671), quality control (due to low call rate) (rs1229984), violation of HWE assumptions (rs1614972, p = 1.6*10^-9^), high missing rate (rs1042026), or high pair-wise correlation with another instrumental SNP (rs1693482, r^2^ = 0.96).

**References**

1. Gelernter J, Kranzler HR, Sherva R, Almasy L, Koesterer R, Smith AH, et al. Genome-wide association study of alcohol dependence:significant findings in African- and European-Americans including novel risk loci. Mol Psychiatry. 2014;19(1):41-9.

2. Zuccolo L, Fitz-Simon N, Gray R, Ring SM, Sayal K, Smith GD, et al. A non-synonymous variant in ADH1B is strongly associated with prenatal alcohol use in a European sample of pregnant women. Hum Mol Genet. 2009;18(22):4457-66.

3. Macgregor S, Lind PA, Bucholz KK, Hansell NK, Madden PA, Richter MM, et al. Associations of ADH and ALDH2 gene variation with self report alcohol reactions, consumption and dependence: an integrated analysis. Hum Mol Genet. 2009;18(3):580-93.

4. Agrawal A, Bierut LJ. Identifying genetic variation for alcohol dependence. Alcohol Res. 2012;34(3):274-81.

5. Ferrari P, McKay JD, Jenab M, Brennan P, Canzian F, Vogel U, et al. Alcohol dehydrogenase and aldehyde dehydrogenase gene polymorphisms, alcohol intake and the risk of colorectal cancer in the European Prospective Investigation into Cancer and Nutrition study. Eur J Clin Nutr. 2012;66(12):1303-8.

6. Li D, Zhao H, Gelernter J. Strong association of the alcohol dehydrogenase 1B gene (ADH1B) with alcohol dependence and alcohol-induced medical diseases. Biol Psychiatry. 2011;70(6):504-12.

7. Bierut LJ, Goate AM, Breslau N, Johnson EO, Bertelsen S, Fox L, et al. ADH1B is associated with alcohol dependence and alcohol consumption in populations of European and African ancestry. Mol Psychiatry. 2012;17(4):445-50.

8. Way M, McQuillin A, Saini J, Ruparelia K, Lydall GJ, Guerrini I, et al. Genetic variants in or near ADH1B and ADH1C affect susceptibility to alcohol dependence in a British and Irish population. Addict Biol. 2015;20(3):594-604.

9. Agrawal A, Verweij KJ, Gillespie NA, Heath AC, Lessov-Schlaggar CN, Martin NG, et al. The genetics of addiction-a translational perspective. Translational psychiatry. 2012;2:e140.

10. Toth R, Fiatal S, Petrovski B, McKee M, Adany R. Combined effect of ADH1B RS1229984, RS2066702 and ADH1C RS1693482/ RS698 alleles on alcoholism and chronic liver diseases. Dis Markers. 2011;31(5):267-77.

11. Li D, Zhao H, Gelernter J. Further clarification of the contribution of the ADH1C gene to vulnerability of alcoholism and selected liver diseases. Hum Genet. 2012;131(8):1361-74.

12. Biernacka JM, Geske JR, Schneekloth TD, Frye MA, Cunningham JM, Choi DS, et al. Replication of genome wide association studies of alcohol dependence: support for association with variation in ADH1C. PLoS One. 2013;8(3):e58798.

13. Norden-Krichmar TM, Gizer IR, Wilhelmsen KC, Schork NJ, Ehlers CL. Protective variant associated with alcohol dependence in a Mexican American cohort. BMC Med Genet. 2014;15:136.

14. Luo X, Kranzler HR, Zuo L, Yang BZ, Lappalainen J, Gelernter J. ADH4 gene variation is associated with alcohol and drug dependence: results from family controlled and population-structured association studies. Pharmacogenet Genomics. 2005;15(11):755-68.

15. Rietschel M, Treutlein J. The genetics of alcohol dependence. Ann N Y Acad Sci. 2013;1282:39-70.
