# Supplementary material for: Causal Role of Alcohol Consumption in an Improved Lipid Profile: The Atherosclerosis Risk in Communities (ARIC) Study
Source: PLoS One. 2016 Feb 5;11(2):e0148765. doi: 10.1371/journal.pone.0148765 (PMC4744040; doi:10.1371/journal.pone.0148765)
Supplement: S2 Table — (DOCX) [file pone.0148765.s002.docx]

**Supporting information**

**Causal role of alcohol consumption in an improved lipid profile: the Atherosclerosis Risk in Communities (ARIC) study**

Khanh N. Vu^1^, Christie M. Ballantyne^2,3^_,_ Ron C. Hoogeveen^2,3^, Vijay Nambi^2,3,4^, Kelly A. Volcik^5^, Eric Boerwinkle^1,6^ Alanna C. Morrison^1*^

^1^School of Public Health, University of Texas Health Science Center at Houston, Houston, TX, USA

^2^Section of Cardiovascular Research, Baylor College of Medicine, Houston, TX, USA

^3^Houston Methodist Debakey Heart and Vascular Center, Houston, TX, USA

^4^Michael E DeBakey Veterans Affairs Hospital, Houston, TX, USA

^5^Department of Biochemistry and Molecular Biology, University of Texas Medical School at Houston, Houston, TX, USA

^6^The Human Genome Sequencing Center, Baylor College of Medicine, Houston, TX, USA

*Corresponding author

E-mail: Alanna.C.Morrison@uth.tmc.edu (ACM)

**S2 Table. Association between unweighted genetic score and alcohol consumption**

| Alcohol consumption | β | 95% CI | | p |
| --- | --- | --- | --- | --- |
| At baseline | 0.06 | 0.03 | 0.09 | **<0.001** |
| At visit 4 | 0.04 | 0.01 | 0.08 | **0.013** |
