# Supplementary material for: Causal Role of Alcohol Consumption in an Improved Lipid Profile: The Atherosclerosis Risk in Communities (ARIC) Study
Source: PLoS One. 2016 Feb 5;11(2):e0148765. doi: 10.1371/journal.pone.0148765 (PMC4744040; doi:10.1371/journal.pone.0148765)
Supplement: S3 Table — (DOCX) [file pone.0148765.s003.docx]

**Supporting information**

**Causal role of alcohol consumption in an improved lipid profile: the Atherosclerosis Risk in Communities (ARIC) study**

Khanh N. Vu^1^, Christie M. Ballantyne^2,3^_,_ Ron C. Hoogeveen^2,3^, Vijay Nambi^2,3,4^, Kelly A. Volcik^5^, Eric Boerwinkle^1,6^ Alanna C. Morrison^1*^

^1^School of Public Health, University of Texas Health Science Center at Houston, Houston, TX, USA

^2^Section of Cardiovascular Research, Baylor College of Medicine, Houston, TX, USA

^3^Houston Methodist Debakey Heart and Vascular Center, Houston, TX, USA

^4^Michael E DeBakey Veterans Affairs Hospital, Houston, TX, USA

^5^Department of Biochemistry and Molecular Biology, University of Texas Medical School at Houston, Houston, TX, USA

^6^The Human Genome Sequencing Center, Baylor College of Medicine, Houston, TX, USA

*Corresponding author

E-mail: Alanna.C.Morrison@uth.tmc.edu (ACM)

**S3 Table. Sensitivity IV analysis excluding never drinkers**

| Lipids | N | Predicted alcohol consumption quartiles | β* | 95% CI | | p^a^ | p overall^b^ | 1^st^-stage partial R^2^ | 1^st^-stage F-value |
| --- | --- | --- | --- | --- | --- | --- | --- | --- | --- |
| TG ¥ | 8,082 | q1 | 0.00 |  |  |  | **<0.001** | 0.17% | 13.98 |
|  |  | q2 | -0.07 | -0.11 | -0.03 | **<0.001** |  |  |  |
|  |  | q3 | -0.14 | -0.21 | -0.07 | **<0.001** |  |  |  |
|  |  | q4 | -0.09 | -0.18 | 0.00 | 0.050 |  |  |  |
| Total cholesterol | 7,959 | q1 | 0.00 |  |  |  | **<0.001** | 0.15% | 12.09 |
|  |  | q2 | -4.39 | -7.44 | -1.33 | **0.005** |  |  |  |
|  |  | q3 | -6.56 | -12.80 | -0.33 | **0.039** |  |  |  |
|  |  | q4 | -3.24 | -10.80 | 4.32 | 0.401 |  |  |  |
| HDL-c ¥ | 8,275 | q1 | 0.00 |  |  |  | 0.181 | 0.18% | 15.05 |
|  |  | q2 | 0.02 | -0.01 | 0.04 | 0.158 |  |  |  |
|  |  | q3 | 0.03 | -0.01 | 0.07 | 0.137 |  |  |  |
|  |  | q4 | 0.02 | -0.03 | 0.08 | 0.359 |  |  |  |
| HDL2-c ¥ | 8,266 | q1 | 0.00 |  |  |  | **0.004** | 0.18% | 15.04 |
|  |  | q2 | 0.05 | 0.01 | 0.09 | **0.022** |  |  |  |
|  |  | q3 | 0.09 | 0.01 | 0.16 | **0.034** |  |  |  |
|  |  | q4 | 0.06 | -0.04 | 0.15 | 0.262 |  |  |  |
| HDL3-c | 8,266 | q1 | 0.00 |  |  |  | 0.985 | 0.18% | 15.04 |
|  |  | q2 | 0.11 | -0.67 | 0.88 | 0.791 |  |  |  |
|  |  | q3 | 0.17 | -1.29 | 1.63 | 0.817 |  |  |  |
|  |  | q4 | 0.30 | -1.51 | 2.10 | 0.745 |  |  |  |
| LDL-c | 7,959 | q1 | 0.00 |  |  |  | **0.002** | 0.15% | 12.09 |
|  |  | q2 | -3.70 | -6.65 | -0.75 | **0.014** |  |  |  |
|  |  | q3 | -5.50 | -11.56 | 0.56 | 0.075 |  |  |  |
|  |  | q4 | -3.08 | -10.38 | 4.23 | 0.409 |  |  |  |
| sdLDL-c ¥# | 7,022 | q1 | 0.00 |  |  |  | **0.003** | 0.11% | 7.44 |
|  |  | q2 | -0.07 | -0.11 | -0.03 | **<0.001** |  |  |  |
|  |  | q3 | -0.10 | -0.18 | -0.03 | **0.009** |  |  |  |
|  |  | q4 | -0.12 | -0.21 | -0.02 | **0.017** |  |  |  |
| apoB ¥# | 6,629 | q1 | 0.00 |  |  |  | **0.004** | 0.12% | 7.86 |
|  |  | q2 | -0.03 | -0.05 | -0.01 | **0.001** |  |  |  |
|  |  | q3 | -0.04 | -0.08 | 0.00 | 0.072 |  |  |  |
|  |  | q4 | -0.04 | -0.08 | 0.01 | 0.156 |  |  |  |
| Lp(a) ¥ | 8,099 | q1 | 0.00 |  |  |  | 0.283 | 0.19% | 15.09 |
|  |  | q2 | 0.02 | -0.06 | 0.11 | 0.601 |  |  |  |
|  |  | q3 | -0.03 | -0.19 | 0.14 | 0.727 |  |  |  |
|  |  | q4 | 0.05 | -0.16 | 0.25 | 0.666 |  |  |  |

*second stage regression coefficient between lipid measures and predicted alcohol consumption quartiles with quartile 1 as the reference group, ^a^Wald p-value comparing each quartile with the quartile 1, ^b^Wald p-value for overall effect of alcohol consumption, ¥ ln transformed, # measured at visit 4
