# Supplementary material for: Causal Role of Alcohol Consumption in an Improved Lipid Profile: The Atherosclerosis Risk in Communities (ARIC) Study
Source: PLoS One. 2016 Feb 5;11(2):e0148765. doi: 10.1371/journal.pone.0148765 (PMC4744040; doi:10.1371/journal.pone.0148765)
Supplement: S4 Table — (DOCX) [file pone.0148765.s004.docx]

**Supporting information**

**Causal role of alcohol consumption in an improved lipid profile: the Atherosclerosis Risk in Communities (ARIC) study**

Khanh N. Vu^1^, Christie M. Ballantyne^2,3^_,_ Ron C. Hoogeveen^2,3^, Vijay Nambi^2,3,4^, Kelly A. Volcik^5^, Eric Boerwinkle^1,6^ Alanna C. Morrison^1*^

^1^School of Public Health, University of Texas Health Science Center at Houston, Houston, TX, USA

^2^Section of Cardiovascular Research, Baylor College of Medicine, Houston, TX, USA

^3^Houston Methodist Debakey Heart and Vascular Center, Houston, TX, USA

^4^Michael E DeBakey Veterans Affairs Hospital, Houston, TX, USA

^5^Department of Biochemistry and Molecular Biology, University of Texas Medical School at Houston, Houston, TX, USA

^6^The Human Genome Sequencing Center, Baylor College of Medicine, Houston, TX, USA

*Corresponding author

E-mail: Alanna.C.Morrison@uth.tmc.edu (ACM)

**S4 Table. Sensitivity IV analysis excluding never and heavy drinkers**

| Lipids | N | Predicted alcohol consumption quartiles | β* | 95% CI | | p^a^ | p overall^b^ | 1^st^-stage partial R^2^ | 1^st^-stage F-value |
| --- | --- | --- | --- | --- | --- | --- | --- | --- | --- |
| TG ¥ | 7,215 | q1 | 0.00 |  |  |  | **<0.001** | 0.15% | 11.16 |
|  |  | q2 | -0.08 | -0.12 | -0.04 | **<0.001** |  |  |  |
|  |  | q3 | -0.16 | -0.24 | -0.09 | **<0.001** |  |  |  |
|  |  | q4 | -0.12 | -0.22 | -0.02 | **0.014** |  |  |  |
| Total cholesterol | 7,105 | q1 | 0.00 |  |  |  | **<0.001** | 0.14% | 9.93 |
|  |  | q2 | -4.12 | -7.31 | -0.93 | **0.011** |  |  |  |
|  |  | q3 | -4.76 | -11.67 | 2.15 | 0.177 |  |  |  |
|  |  | q4 | -0.85 | -9.09 | 7.39 | 0.840 |  |  |  |
| HDL-c ¥ | 7,391 | q1 | 0.00 |  |  |  | 0.130 | 0.16% | 11.95 |
|  |  | q2 | 0.02 | 0.00 | 0.05 | **0.049** |  |  |  |
|  |  | q3 | 0.03 | -0.01 | 0.08 | 0.138 |  |  |  |
|  |  | q4 | 0.03 | -0.02 | 0.08 | 0.287 |  |  |  |
| HDL2-c ¥ | 7,382 | q1 | 0.00 |  |  |  | **0.008** | 0.16% | 11.93 |
|  |  | q2 | 0.05 | 0.01 | 0.09 | **0.014** |  |  |  |
|  |  | q3 | 0.07 | -0.01 | 0.16 | 0.089 |  |  |  |
|  |  | q4 | 0.05 | -0.06 | 0.15 | 0.382 |  |  |  |
| HDL3-c | 7,382 | q1 | 0.00 |  |  |  | 0.776 | 0.16% | 11.93 |
|  |  | q2 | 0.39 | -0.40 | 1.18 | 0.334 |  |  |  |
|  |  | q3 | 0.42 | -1.08 | 1.93 | 0.580 |  |  |  |
|  |  | q4 | 0.68 | -1.18 | 2.53 | 0.475 |  |  |  |
| LDL-c | 7,105 | q1 | 0.00 |  |  |  | **0.005** | 0.14% | 9.93 |
|  |  | q2 | -3.44 | -6.50 | -0.38 | **0.027** |  |  |  |
|  |  | q3 | -2.93 | -9.56 | 3.71 | 0.387 |  |  |  |
|  |  | q4 | -0.04 | -7.93 | 7.85 | 0.992 |  |  |  |
| sdLDL-c ¥# | 6,440 | q1 | 0.00 |  |  |  | **0.007** | 0.10% | 6.43 |
|  |  | q2 | -0.06 | -0.10 | -0.02 | **0.002** |  |  |  |
|  |  | q3 | -0.12 | -0.20 | -0.03 | **0.006** |  |  |  |
|  |  | q4 | -0.13 | -0.23 | -0.03 | **0.014** |  |  |  |
| apoB ¥# | 6,079 | q1 | 0.00 |  |  |  | **0.010** | 0.11% | 6.74 |
|  |  | q2 | -0.03 | -0.05 | -0.01 | **0.002** |  |  |  |
|  |  | q3 | -0.04 | -0.08 | 0.00 | 0.079 |  |  |  |
|  |  | q4 | -0.04 | -0.09 | 0.02 | 0.180 |  |  |  |
| Lp(a) ¥ | 7,235 | q1 | 0.00 |  |  |  | 0.384 | 0.16% | 11.67 |
|  |  | q2 | 0.03 | -0.06 | 0.12 | 0.530 |  |  |  |
|  |  | q3 | -0.03 | -0.21 | 0.15 | 0.755 |  |  |  |
|  |  | q4 | 0.04 | -0.18 | 0.26 | 0.717 |  |  |  |

*second stage regression coefficient between lipid measures and predicted alcohol consumption quartiles with quartile 1 as the reference group, ^a^Wald p-value comparing each quartile with the quartile 1, ^b^Wald p-value for overall effect of alcohol consumption, ¥ ln transformed, # measured at visit 4
