# Supplementary material for: Causal Role of Alcohol Consumption in an Improved Lipid Profile: The Atherosclerosis Risk in Communities (ARIC) Study
Source: PLoS One. 2016 Feb 5;11(2):e0148765. doi: 10.1371/journal.pone.0148765 (PMC4744040; doi:10.1371/journal.pone.0148765)
Supplement: S5 Table — (DOCX) [file pone.0148765.s005.docx]

**Supporting information**

**Causal role of alcohol consumption in an improved lipid profile: the Atherosclerosis Risk in Communities (ARIC) study**

Khanh N. Vu^1^, Christie M. Ballantyne^2,3^_,_ Ron C. Hoogeveen^2,3^, Vijay Nambi^2,3,4^, Kelly A. Volcik^5^, Eric Boerwinkle^1,6^ Alanna C. Morrison^1*^

^1^School of Public Health, University of Texas Health Science Center at Houston, Houston, TX, USA

^2^Section of Cardiovascular Research, Baylor College of Medicine, Houston, TX, USA

^3^Houston Methodist Debakey Heart and Vascular Center, Houston, TX, USA

^4^Michael E DeBakey Veterans Affairs Hospital, Houston, TX, USA

^5^Department of Biochemistry and Molecular Biology, University of Texas Medical School at Houston, Houston, TX, USA

^6^The Human Genome Sequencing Center, Baylor College of Medicine, Houston, TX, USA

*Corresponding author

E-mail: Alanna.C.Morrison@uth.tmc.edu (ACM)

**S5 Table. Sensitivity IV analysis excluding never and former drinkers**

| Lipids | N | Predicted alcohol consumption quartiles | β* | 95% CI | | p^a^ | p overall^b^ | 1^st^-stage partial R^2^ | 1^st^-stage F-value |
| --- | --- | --- | --- | --- | --- | --- | --- | --- | --- |
| TG ¥ | 6,420 | q1 | 0.00 |  |  |  | **<0.001** | 0.18% | 11.56 |
|  |  | q2 | -0.05 | -0.09 | -0.01 | **0.011** |  |  |  |
|  |  | q3 | -0.10 | -0.18 | -0.03 | **0.008** |  |  |  |
|  |  | q4 | -0.05 | -0.15 | 0.05 | 0.313 |  |  |  |
| Total cholesterol | 6,328 | q1 | 0.00 |  |  |  | **0.001** | 0.15% | 9.89 |
|  |  | q2 | -4.24 | -7.64 | -0.84 | **0.014** |  |  |  |
|  |  | q3 | -4.38 | -11.29 | 2.53 | 0.214 |  |  |  |
|  |  | q4 | -0.91 | -9.36 | 7.54 | 0.833 |  |  |  |
| HDL-c ¥ | 6,567 | q1 | 0.00 |  |  |  | 0.832 | 0.19% | 12.85 |
|  |  | q2 | 0.01 | -0.02 | 0.03 | 0.648 |  |  |  |
|  |  | q3 | 0.01 | -0.03 | 0.06 | 0.612 |  |  |  |
|  |  | q4 | 0.01 | -0.05 | 0.06 | 0.842 |  |  |  |
| HDL2-c ¥ | 6,561 | q1 | 0.00 |  |  |  | 0.072 | 0.19% | 12.87 |
|  |  | q2 | 0.03 | -0.01 | 0.08 | 0.139 |  |  |  |
|  |  | q3 | 0.07 | -0.02 | 0.15 | 0.122 |  |  |  |
|  |  | q4 | 0.04 | -0.07 | 0.14 | 0.483 |  |  |  |
| HDL3-c | 6,561 | q1 | 0.00 |  |  |  | 0.896 | 0.19% | 12.87 |
|  |  | q2 | -0.18 | -1.05 | 0.69 | 0.687 |  |  |  |
|  |  | q3 | -0.44 | -2.04 | 1.16 | 0.593 |  |  |  |
|  |  | q4 | -0.30 | -2.29 | 1.70 | 0.769 |  |  |  |
| LDL-c | 6,328 | q1 | 0.00 |  |  |  | **0.027** | 0.15% | 9.89 |
|  |  | q2 | -3.26 | -6.54 | 0.03 | 0.052 |  |  |  |
|  |  | q3 | -2.73 | -9.48 | 4.02 | 0.428 |  |  |  |
|  |  | q4 | -0.28 | -8.47 | 7.91 | 0.947 |  |  |  |
| sdLDL-c ¥# | 4,518 | q1 | 0.00 |  |  |  | **0.016** | 0.11% | 5.01 |
|  |  | q2 | -0.07 | -0.12 | -0.02 | **0.003** |  |  |  |
|  |  | q3 | -0.12 | -0.21 | -0.02 | **0.014** |  |  |  |
|  |  | q4 | -0.15 | -0.26 | -0.03 | **0.013** |  |  |  |
| apoB ¥# | 4,262 | q1 | 0.00 |  |  |  | **0.040** | 0.11% | 4.65 |
|  |  | q2 | -0.03 | -0.06 | -0.01 | **0.008** |  |  |  |
|  |  | q3 | -0.03 | -0.08 | 0.02 | 0.189 |  |  |  |
|  |  | q4 | -0.03 | -0.09 | 0.03 | 0.327 |  |  |  |
| Lp(a) ¥ | 6,430 | q1 | 0.00 |  |  |  | 0.550 | 0.20% | 12.87 |
|  |  | q2 | 0.02 | -0.08 | 0.12 | 0.733 |  |  |  |
|  |  | q3 | 0.01 | -0.17 | 0.19 | 0.900 |  |  |  |
|  |  | q4 | 0.08 | -0.15 | 0.31 | 0.479 |  |  |  |

*second stage regression coefficient between lipid measures and predicted alcohol consumption quartiles with quartile 1 as the reference group, ^a^Wald p-value comparing each quartile with the quartile 1, ^b^Wald p-value for overall effect of alcohol consumption, ¥ ln transformed, # measured at visit 4
